# Genomic Predictions and Genome-Wide Association Study of Resistance Against *Piscirickettsia salmonis* in Coho Salmon (*Oncorhynchus kisutch*) Using ddRAD Sequencing

**DOI:** 10.1534/g3.118.200053

**Published:** 2018-02-22

**Authors:** Agustín Barría, Kris A. Christensen, Grazyella M. Yoshida, Katharina Correa, Ana Jedlicki, Jean P. Lhorente, William S. Davidson, José M. Yáñez

**Affiliations:** * Facultad de Ciencias Veterinarias y Pecuarias, Universidad de Chile, La Pintana, Santiago 8820808, Chile; † Doctorado en Acuicultura, Programa Cooperativo Universidad de Chile, Universidad Católica del Norte, Pontificia Universidad Católica de Valparaíso, 8820808 Chile; ‡ Department of Molecular Biology and Biochemistry, Simon Fraser University, Burnaby, BC V5A 1S6, Canada; § Animal Science Department, Universidade Estadual Paulista “Júlio de Mesquita Filho”, Faculdade de Ciências Agrárias e Veterinárias, Campus Jaboticabal, Jaboticabal 14884-900, Brazil; ** Aquainnovo S.A., Puerto Montt 5503032, Chile; †† Núcleo Milenio INVASAL, Concepción 4070386, Chile

**Keywords:** selective breeding, genotyping by sequencing, *Oncorhynchus kisutch*, disease resistance, GWAS, Genomic Selection, GenPred, Shared Data Resources

## Abstract

*Piscirickettsia salmonis* is one of the main infectious diseases affecting coho salmon (*Oncorhynchus kisutch*) farming, and current treatments have been ineffective for the control of this disease. Genetic improvement for *P. salmonis* resistance has been proposed as a feasible alternative for the control of this infectious disease in farmed fish. Genotyping by sequencing (GBS) strategies allow genotyping of hundreds of individuals with thousands of single nucleotide polymorphisms (SNPs), which can be used to perform genome wide association studies (GWAS) and predict genetic values using genome-wide information. We used double-digest restriction-site associated DNA (ddRAD) sequencing to dissect the genetic architecture of resistance against *P. salmonis* in a farmed coho salmon population and to identify molecular markers associated with the trait. We also evaluated genomic selection (GS) models in order to determine the potential to accelerate the genetic improvement of this trait by means of using genome-wide molecular information. A total of 764 individuals from 33 full-sib families (17 highly resistant and 16 highly susceptible) were experimentally challenged against *P. salmonis* and their genotypes were assayed using ddRAD sequencing. A total of 9,389 SNPs markers were identified in the population. These markers were used to test genomic selection models and compare different GWAS methodologies for resistance measured as day of death (DD) and binary survival (BIN). Genomic selection models showed higher accuracies than the traditional pedigree-based best linear unbiased prediction (PBLUP) method, for both DD and BIN. The models showed an improvement of up to 95% and 155% respectively over PBLUP. One SNP related with B-cell development was identified as a potential functional candidate associated with resistance to *P. salmonis* defined as DD.

Chile is the largest producer of coho salmon (*Oncorhynchus kisutch*) globally, reaching about 160,000 tons in 2014, representing more than 90% of total production (FAO 2016). However, the success and sustainability of this industry is constantly threatened by infectious diseases, including Salmon Rickettsial Syndrome (SRS). This disease is caused by *Piscirickettsia salmonis*, a gram-negative and facultative intracellular bacteria, which was isolated for the first time in Chile in coho salmon (Cvitanich *et al.* 1991). Data from the Chilean National Fisheries and Aquaculture Service (Sernapesca) indicates that during the first half of 2016, 53% of the moralities ascribed to infectious diseases in coho salmon were associated with SRS (Sernapesca 2016). To date, control measures and treatments for SRS are based on antibiotics and vaccines. However, both strategies have not had the expected effectiveness under field conditions (Rozas and Enríquez 2014).

Because of this, it is necessary to develop alternative strategies for the control of this disease (Yáñez *et al.* 2014a). In this regard, breeding for enhanced disease resistance is a feasible and sustainable option to improve animal health, welfare and productivity (Stear *et al.* 2001). A primary requisite for including disease resistance into a breeding program is the presence of significant additive genetic variation for the trait (Falconer and Mackay 1996). Commonly, data to evaluate resistance comes from experimental challenges carried out using siblings of the selection candidates (Ødegård *et al.* 2011; Yáñez *et al.* 2014a). Quantitative studies have estimated significant genetic variation for resistance against different pathogens in salmonid species (Ødegård *et al.* 2011; Yáñez *et al.* 2014a). For instance, low to moderate heritabilities for resistance against *P. salmonis* in Atlantic salmon (*Salmo salar*) (h^2^ = 0.11 to 0.41) (Yáñez *et al.* 2013; Yáñez *et al.* 2014b) and coho salmon (h^2^ = 0.16) (Yáñez *et al.* 2016a) have been estimated.

Marker assisted selection (MAS) can improve production traits in cases where the phenotypes are difficult to measure in the selected candidates (*e.g.*, disease resistance traits) and the total additive genetic variance explained by genetic markers is high (Hayes and Goddard 2010). This methodology has been successfully applied for the improvement of resistance against the Infectious Pancreatic Necrosis Virus (IPNV) in Atlantic salmon, which is controlled by a major quantitative trait locus (QTL) (Houston *et al.* 2012; Moen *et al.* 2015). In the case of polygenic traits, genomic selection (GS) (Meuwissen *et al.* 2001) can significantly improve selection accuracy of breeding values compared to traditional selection, and therefore enhance the response of selection for disease resistance in salmonid species (Tsai *et al.* 2016; Vallejo *et al.* 2016, 2017a; Bangera *et al.* 2017; Correa *et al.* 2017; Yoshida *et al.* 2017).

Genotyping by sequencing (GBS) is an alternative for genotyping in cases when SNP panels are not available. This approach reduces the complexity of the genome, and can be used to identify thousands of markers without prior marker discovery efforts or a reference genome. Currently, several approaches of GBS have been developed, significantly reducing the cost and labor (Baird *et al.* 2008; Elshire *et al.* 2011; Peterson *et al.* 2012). These methodologies have been widely used in salmonid species, to generate dense linkage maps (Brieuc *et al.* 2014; Gonen *et al.* 2014), perform association studies to identify genomic regions involved in the resistance against pathogens (Campbell *et al.* 2014; Liu *et al.* 2015; Palti *et al.* 2015b) and generate SNPs resources (Houston *et al.* 2012).

Double-digest restriction-site associated DNA (ddRAD) reduces DNA complexity by digesting DNA with two restriction enzymes (REs) simultaneously, without random shearing (Peterson *et al.* 2012). This approach has been widely used in genetic studies in aquaculture species (reviewed in Robledo *et al.* 2017).

In the present study, we used ddRAD sequencing to dissect the genetic architecture of resistance against *P. salmonis* in a farmed coho salmon population and identify molecular markers associated with the trait. Furthermore, GS models were used to evaluate the potential to accelerate the genetic improvement of resistance against *P. salmonis* in this coho salmon population by means of using genome-wide molecular information.

## Materials and Methods

### Coho salmon breeding population

The coho salmon population used in the present study came from a unique 2012 year-class population. This population belongs to a genetic improvement program that was established in 1997 and is owned by Pesquera Antares and managed by Aquainnovo (Puerto Montt, Chile). Further details about this breeding population, in terms of reproductive management, rearing conditions, fish tagging and breeding objectives are described by Yáñez *et al.* (2014c; 2016a) and Dufflocq *et al.* (2016).

### Experimental challenge

The experimental challenge against *P. salmonis* was performed as it is described in detail by Correa *et al.* (2015a) and Yáñez *et al.* (2016a). Briefly, 2,606 individuals, belonging to 107 maternal full-sib families and 60 paternal half-sib families, were challenged against *P. salmonis*. Prior to the experiment, each fish was tagged with a passive integrated transponder (PIT-tag), placed in the abdominal cavity for genealogy traceability during the challenge test. The *P. salmonis* challenge was performed at Aquainnovo´s research station, located in Lenca River, X Region, Chile.

For the lethal dose 50 (LD_50_) calculation, a random sample of 80 fish were selected from the population. Four different dilutions from the *P. salmonis* inoculum were evaluated (1/10, 1/100, 1/1000 and 1/10000). Twenty fish were challenged at each dilution. The dilutions were intraperitoneally (IP) injected with a volume of 0.2ml/fish. Daily mortality was recorded. This preliminary test spanned 26 days and a dilution of 1:680 was estimated as the LD_50_.

For the main challenge, fish were distributed into three tanks (7m^3^) with a salt water concentration of 31 ppt. An average of eight individuals (ranging from 1 to 18) from each of the 107 families were distributed into each tank. The experimental challenge was performed through an intraperitoneal (IP) injection with 0.2ml/fish of the LD_50_ inoculum. The average weight of the fish at the inoculation was 279g (SD = 138g). To ensure that these fish were free from other pathogens, qRT-PCR was previously performed in order to control for the presence of Infectious Salmon Anemia Virus (ISAV), IPNV and *Flavobacterium spp*.

The *P. salmonis* challenge continued for up to 50 days post IP injection. Throughout the challenge, environmental parameters (pH, temperature, salinity and oxygen) were measured and controlled. Fish were removed from the tanks after death, and a sample of the anterior kidney was taken and stored at -80° in RNALater. A necropsy assay was performed in conjunction with qRT-PCR to confirm the cause of death and the presence of *P. salmonis*. This was also done to control for the presence of other pathogens, such as *Vibrio ordalii*, *Renibacterium salmoninarum* and IPNV.

### ddRAD library preparation and sequencing

Ten ddRAD libraries were produced by multiplexing 828 individuals following the protocol described by Peterson *et al.* (2012). For this, 64 parents (males and females) and 764 offspring representing the 17 most resistant and 16 most susceptible families were selected. An average of 23 (ranging from 11 to 43 individuals) offspring per family were chosen. Briefly, total DNA was extracted using the commercial kit Wizard SV Genomic DNA purification System (Promega) according to the manufacturer’s protocol. Between 80 and 200 ng of DNA, from each individual was digested with two restriction enzymes (New England Biolabs, UK; NEB); 10 U of *Sbf*I (specific for the CCTGCA|GG motif)) and *Mse*I (specific for the T|TAA motif) in a 12 µl reaction volume, including 1 µl of *Sbf*I and *Mse*I adapter (8.3 pM), for 90 min at 37°. The ligation reaction was carried out by adding 1 µl of T4 ligase (NEB) diluted 1:100 in T4 buffer and incubating for 150 min at 37° and subsequently at 16° overnight.

Each ligation mix was diluted with 189 µl of dilute TE buffer (1:10). Kodak DNA Polymerase (ABM), a high-fidelity polymerase, was used to amplify DNA fragments with the correct adapters. PCR reactions (20 µl) were prepared containing 10 µl of PCR mix 2x, 1 µl of primer mix (10 µM each), 6 µl of diluted ligation mix and 3 µl of nuclease-free water. Each sample was PCR amplified using the following conditions: 95° for 2 min, followed by 17 cycles of 95° for 20s, 66° for 30s and 68° for 40s. After PCR, amplicon quality was checked by loading 5 µl on a 2% agarose gel. Subsequently, samples were pooled, so that the final concentration was similar among them within each library. Each library was concentrated through an evaporation step for 80 min in a Centrivap Mobile Console Centrifugal Evaporator (Labconco). This step was conducted until 300 µl of the generated library was obtained. Final volume of each library was loaded on a 1% agarose gel. Size of the bands selected for sequencing ranged from 750 and 1,500 bp and between 1,800 and 2,500 bp. DNA was purified through the QIAquick gel extraction kit (Qiagen) following manufacturer’s instructions. Finally, libraries were sequenced on an Illumina Hiseq2500 platform, using 150 base single-end.

### SNP identification

Raw sequence reads obtained from Illumina sequencing were analyzed using STACKS v. 1.41 (Catchen *et al.* 2011, 2013). This software was specifically developed to analyze short-read data generated through next generation sequencing (NGS) (Davey *et al.* 2013).

Sample reads were trimmed to 134 bp for all subsequent analyses, demultiplexed and filtered using process_radtags. Rad-tags which passed the quality filter were aligned to the Oncorhynchus kisutch reference genome (GenBank: MPKV00000000.1) using BWA v. 0.7.12 (Li and Durbin 2009). The reference genome was indexed (using the index function) and alignments were performed using the mem algorithm; all parameters were set as default. Loci were then built using pstacks with a minimum depth of coverage of three to build a locus (-m 3).

A catalog of loci was constructed using the cstacks program using only the parents’ loci from pstacks. To build the catalog, the maximum number of mismatches allowed between sample tags was set to three (-n 3), and the matching was based on genomic location (g). After catalog construction, the sstacks program was used in order to match rad-tags against the catalog based again on genomic location (g), followed by the populations software, using defaults parameters. Loci were considered as valid if they were present in at least 75% of the individuals of the population. As a quality control step, the following parameters were used to filter low-confidence SNPs: Minor Allele Frequency (MAF) ≤ 0.05, Hardy-Weinberg Equilibrium (HWE) *P* < 1x10-6 and genotyping call rate < 0.80. Individuals were removed from the analyses if their genotyping call rates were below 0.70.

### Trait definitions

Resistance against *P. salmonis* was defined as the day of death (DD) with values ranging from 1 to 50 depending on the time of death. Additionally, resistance was also evaluated as a binary (BIN) trait, either dead or alive at the end of the challenge. Values for this trait were 1 in cases where the fish died during the challenge, or 0 if the fish survived until the end of the challenge. Initial Body Weight (IW) for each fish, was measured prior to the IP injection.

### Pedigree-based BLUP

All challenged individuals (n = 2,606) were used for the pedigree-based approach, PBLUP, as a control for the performance evaluation of genomic predictions. A linear univariate animal model was used to estimate variance components and predict Estimated Breeding Values (EBVs) for DD, while for BIN a univariate threshold animal model was fitted (Table 1). The model used was as follows:

**Table 1:** Estimated genetic parameters and accuracy of breeding values (EBV) estimation for resistance against *P. salmonis* using a pedigree-based model

**Phenotype***^**a**^*	$\sigma_{a}^{2}$	$\sigma_{e}^{2}$	**h^2^(SE)**	**Accuracy (R)**
DD	12.55	77.60	0.14(0.034)	0.271
BIN	0.38	1.00	0.27 (0.043)	0.316

a The BLUP analysis included the phenotype of all the progeny of 107 families challenged against *Piscirickettsia salmonis* (n = 2,606)

y=Xβ+Tp+e 

In the previous equation, y is a vector of phenotypes (BIN or DD), β is a vector of fixed effects (sex and tank as factors, and initial weight as covariate), p is a vector of random additive polygenic genetic effects that follows a normal distribution ∼N(0,A$\sigma_{p}^{2}$), X and T are incidence matrices, A is the additive relationship matrix, and e is the random residual (Lynch and Walsh 1998). Both models were fitted using the BLUPF90 set of programs (Misztal *et al.* 2016) by means of the AIREMLF90 and THRGIBBS1F90 modules to analyze DD and BIN, respectively. The MCMC Gibbs sampling scheme set for running THRGIBBS1F90, included a total of 200,000 iterations. The first 20,000 were discarded as burn-in iterations, and then from the remaining 180,000 samples, one from every 50 samples was saved for analysis. This Gibbs sampling scheme collected 3,600 independent samples for analysis.

Heritabilities for PBLUP models were computed as follows:

$$h_{i}^{2}=\frac{\sigma_{ai}^{2}}{\sigma_{ai}^{2}+\sigma_{ei}^{2}}$$

where $\sigma_{ai}^{2}$ and $\sigma_{ei}^{2}$ are the additive genetic and residual variances for each trait. In the case of BIN, the residual variance was set to 1.

### Genomic BLUP

The SNP based variance components and GEBVs were estimated using genomic BLUP (GBLUP), similar to the PBLUP model described above, and implemented in the BLUPF90 software. The GBLUP is a modification of the PBLUP method, where *g* is a vector of random additive genetic polygenic effects with a distribution ∼N(0, G$\sigma_{g}^{2}$) and the numerator relationship matrix A is replaced by a genomic relationship matrix G, as described by (VanRaden 2008). Only genotyped animals, which passed all quality controls (n = 580) were analyzed.

### Single step genomic GBLUP

The single-step GBLUP (ssGBLUP) and weighted single-step GBLUP (wssGBLUP) models were similar to the PBLUP model except for a combined genomic and pedigree relationship. The kinship matrix used was H (Aguilar *et al.* 2010), in which genotype and pedigree data are combined. The inverse of the matrix H is:

$$H^{-1}=A^{-1}+\left[\begin{array}{cc} 0 & 0\\ 0 & G^{-1}-A_{22}^{-1} \end{array}\right]$$

where $A^{-1}$ is the inverse numerator relationship matrix for all animals, $A_{22}^{-1}$ is the inverse of a pedigree-based relationship matrix for genotyped animals only, and $G^{-1}$ is the inverse genomic relationship matrix. SNPs were equally weighted and given an initial value of one in the ssGBLUP method. In the wssGBLUP method, the marker variances were used as weights. The marker variances were estimated from allele frequencies, and from marker effects that were calculated in the ssGBLUP method (Wang *et al.* 2014). The DD trait was analyzed as a linear trait using AIREMLF90 and BLUPF90, whereas, BIN was analyzed as a threshold trait with THRGIBBS1F90 in the BLUPF90 family of programs (Misztal *et al.* 2016). The MCMC Gibbs scheme for the estimation of the genetic parameters for BIN for ssGBLUP and wssGBLUP, were estimated identically as described above. The ssGBLUP and wssGBLUP models included all the genotyped animals which passed quality control (n = 580), and all the phenotyped fish (n = 2,606) from 107 families.

### Bayes C

The Bayes C (Habier *et al.* 2011) analyses were performed using GS3 software. A total of 200,000 iterations were used in the Gibbs sampling, with a burn-in period of 20,000 cycles. The results were saved every 50 cycles. The number of samples in this analysis totaled 4,000. Convergence and autocorrelation were assessed by visual inspection of trace plots of the posterior variance components. The adjusted model can be described, in matrix notation, as follows:

$$y=X\beta +Tp+\overset{n}{\underset{i=1}{\sum }}g_{i}a_{i}\delta_{i}+e$$

where y is the vector of phenotypic records (DD or BIN), X is an incidence matrix of fixed effects (sex and tank as factors and IW as covariate), β is the vector of fixed effects, T is an incidence matrix of polygenic effects, p is a random vector of polygenic effects of all individuals in the pedigree, $g_{i}$ is the vector of the genotypes for the *i^th^* SNP for each animal, $a_{i}$ is the random allele substitution effect of the *i^th^* SNP, $\delta_{i}$ is an indicator variable (0, 1) sampled from a binomial distribution with parameters determined such that 1% of the markers were included in the model, and e is a vector of residual effects. The following prior distributions were assumed for the genetic random effects: Independent and identical mixture distributions for the SNP effects; each SNP has a point mass at zero having a probability π and a univariate normal distribution with a probability of 1 – π with null mean and variance ${\text{σ}}_{\text{a}}^{2}$; which in turn has a scaled inverse chi-squared prior with ${\text{v}}_{\text{a}}=4$ degrees of freedom and scale parameter ${\text{s}}_{\text{a}}^{2}$ (or ${\text{s}}_{\text{e}}^{2}$) (Fernando and Garrick 2013). The scale parameter was estimated as a function of the genetic variance population, based on the mean SNP allele frequency and number of markers assumed with nonzero effects (Fernando *et al.* 2007). Only genotyped animals, which passed quality control (n = 580) were used.

### Genomic prediction accuracy

The different models were compared using a fivefold cross validation scheme. To reduce stochastic effects of sampling, the cross-validation analysis was replicated ten times. Briefly, all challenged individuals (genotyped, phenotyped, or both), were randomly separated into five validations sets. For each set, predictions were made by masking the animals’ phenotypes and using the remaining fish as a training set to estimate the marker effects. Thus, for each cross-validation run, the dataset was split into a training set (80%) and a validation set (20%). Accuracy was used to assess the performance of each model and was estimated as follows:

$$r_{EBV,BV}=\frac{r_{EBV,y}}{h}$$

where $r_{EBV,y}$ is the correlation between the EBV of a given model (predicted for the validation set using information from the training set) and the actual phenotype, while h is the square root of the pedigree-based estimate of heritability (Correa *et al.* 2017; Legarra 2008; Palaiokostas *et al.* 2016; Tsai *et al.* 2016). Finally, accuracies were calculated for each model and compared to those obtained with the PBLUP model.

### Genome-wide association study

In order to identify associations between genetic markers and *P. salmonis* resistance, as DD or BIN, four genome-wide association methodologies were performed using the BLUPF90 set of programs (Misztal *et al.* 2016). GBLUP, ssGBLUP, wssGBLUP and w3ssGBLUP models were used to analyze the DD and BIN traits, using a linear and a threshold model respectively (as described above in model 1). For the GBLUP model, the pedigree-based relationship matrix (A) was replaced by a genomic matrix (G). For the ssGBLUP, wssGBLUP and w3ssGBLUP GWAS models the H matrix was used as described above. SNPs were weighted equally (and given a weight of one), for the ssGBLUP model. For the wssGBLUP and w3ssGBLUP methods, weights were determined based on individual marker variances that were estimated using both the marker effects, which were calculated previously for the ssGBLUP model, and marker allele frequencies (Wang *et al.* 2014). For the GBLUP GWAS model, only genotyped animals, which passed quality control (n = 580), were analyzed. For the ssGBLUP, wssGBLUP and w3ssGBLUP GWAS models, all the genotyped animals passing quality control (n = 580), and all the phenotyped fish (n = 2,606) were used. Due to practical reasons, the molecular markers anchored to the different scaffolds not placed in chromosomes, were assigned as chromosome 31. The parents of the challenged individuals were not included in the GWAS analysis because they did not have associated phenotype information, as they were not submitted to the challenge experiment.

### Data availability

Table S1 contains genotypic data (available at the public dryad digital repository https://doi.org/10.5061/dryad.b273q6p), Table S2 contains phenotypic data, and Table S3 contains the pedigree information. Table S4 contains the full list of genes located within the top ten 1-Mbp windows proximate to each SNP associated with P. salmonis resistance for DD and BIN identified through wssGBLUP. Table S5 contains information of the top ten markers which explain the highest percentage of the genetic variance for each method and trait. Table S6 contains results from the 10 replicated CV.

## Results

### Challenge test

**Figure 1:**
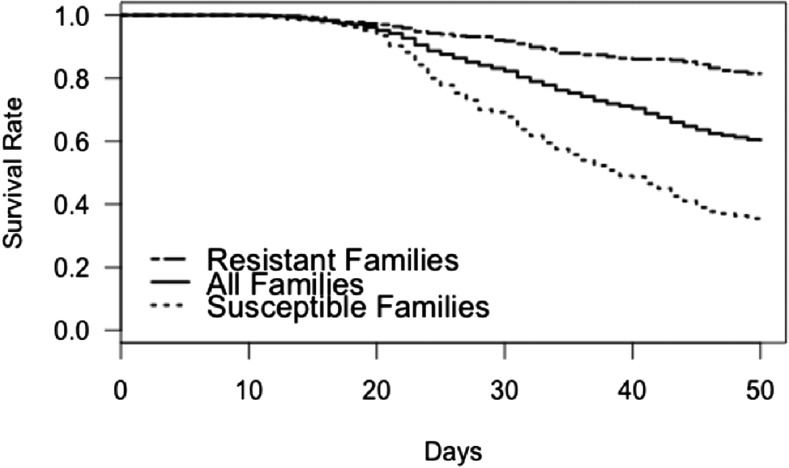
Kaplan-Meier curves for *Piscirickettsia salmonis* experimental challenge in coho salmon. Average mortality curves for the 107 full-sib families, and the 17 best and 16 worst families.

Mortality began on the 10^th^ day after the *P. salmonis* challenge, with evident symptoms of SRS and pathological lesions typical of SRS. These signs include swollen kidney, splenomegaly and yellowish liver tone and coloration (Rozas and Enríquez 2014). Challenged families showed considerable phenotypic variation for *P. salmonis* resistance. Average mortality of all the 107 families reached 38.53% during the 50-day challenge. The average cumulative mortality rate among the 17 best and 16 worst families, selected for genotyping, reached 19% and 63%, respectively (Figure 1).

### ddRAD sequencing

Prior to quality control (QC), per base quality (Phred score) was evaluated. The average quality score ranged from 36 to 38 among libraries, indicating high quality of data. Illumina sequencing, including parents, yielded an average of 156,058,078 (± 16 million) of raw sequences per library. After initial QC, which included the removal of low quality sequences and reads with either missing or ambiguous barcodes, an average of 31,660,024 of the reads were removed. In parallel with QC, reads were trimmed to 134 bp. Thus, 79% of the raw reads were retained for subsequent analysis. To create a set of all possible alleles in the population, data sets of the parental samples were used to create a STACKS catalog. This catalog consisted of 106,309 unique ddRAD loci from which 20,068 markers from 757 individuals were identified. Quality filtering reduced this to 9,389 putative bi allelic SNPs (see Table S1) with an average sequencing depth of 38x ranging from 11x to 501x. These markers were identified segregating along the genome of 580 individuals (see Table S1).

### Genome-wide association analysis

**Figure 2:**
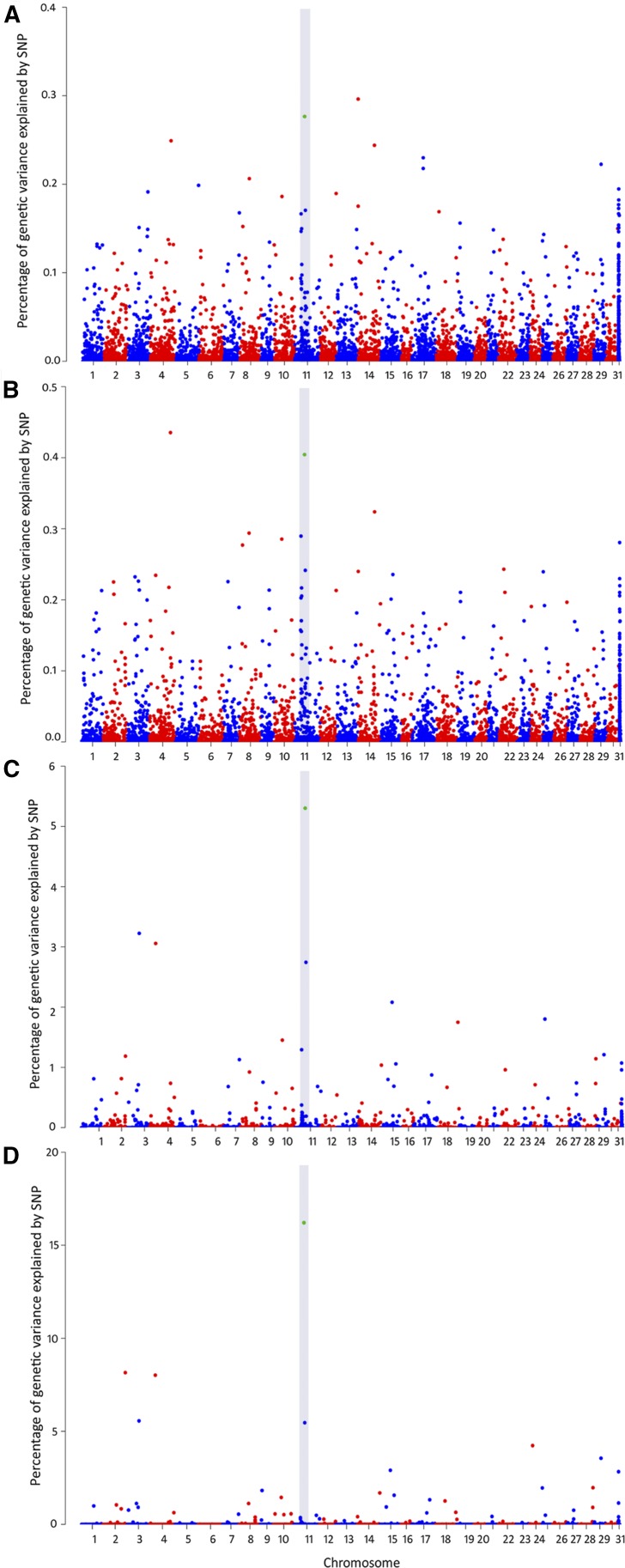
Genomic association analyses for resistance against *Piscirickettsia salmonis* in a coho salmon population (defined as day of death) for four different models; GBLUP (A), ssGBLUP (B), wssGBLUP (C) and w3ssGBLUP (D). The gray area highlights the SNPs (in green), which are among the top ten markers explaining a high percentage of the genetic variance in the four models.

**Figure 3:**
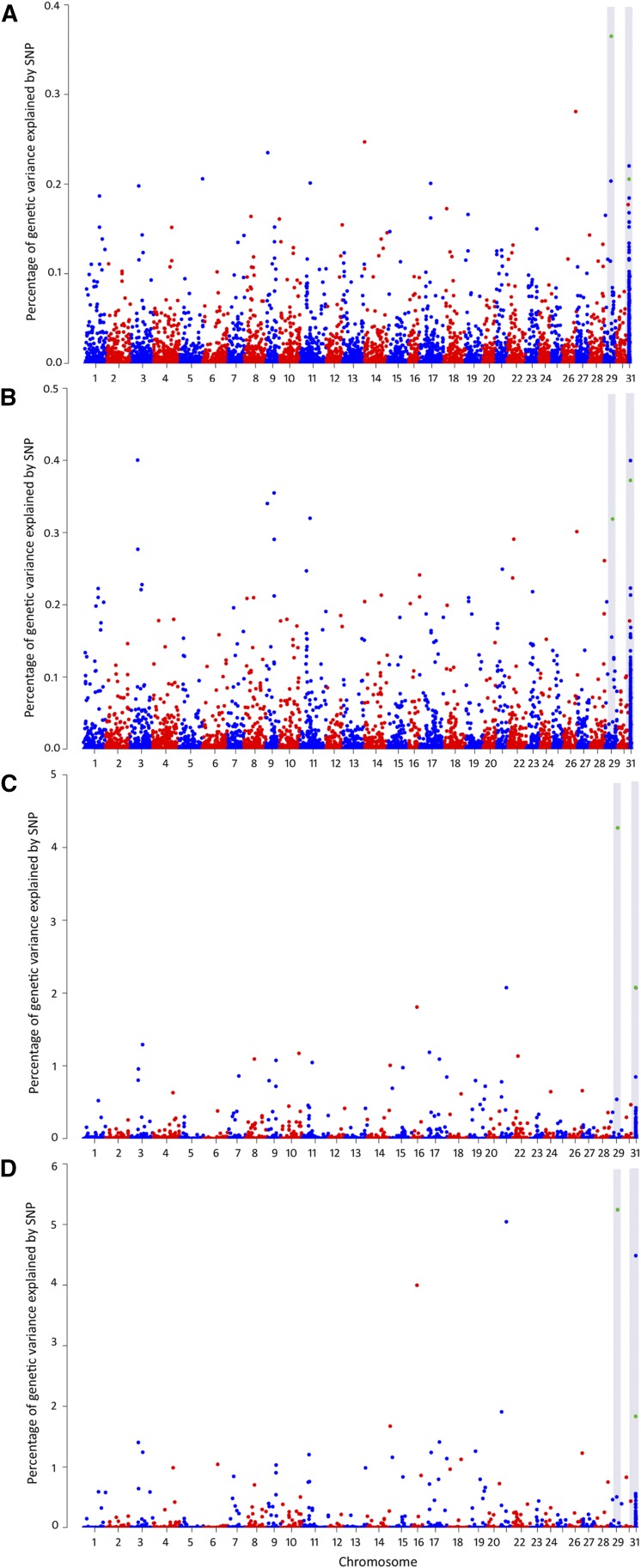
Genomic association analyses for resistance against *Piscirickettsia salmonis* in a coho salmon population (for survival as a binary trait) for four different models; GBLUP (A), ssGBLUP (B), wssGBLUP (C) and w3ssGBLUP (D). The gray area highlights SNPs (in green) that were among the top ten markers explaining a high percentage of the genetic variance in the four models.

Four genome-wide association methodologies were performed either for DD and BIN. These approximations include GBLUP, ssGBLUP, wssGBLUP and w3ssGBLUP. For both traits, all the models showed a similar association pattern. In the case of the ssGBLUP methodology, the GWAS plots become less noisy as the iterations progress, and the peaks associated with the traits become more distinct (Figure 2 and Figure 3). For DD, a marker potentially associated with *P. salmonis* resistance was located on chromosome 11 (Figure 2). This marker was identified in all of the four models and was within the top ten markers explaining most of the percentage of the genetic variance (Table 2).

**Table 2:** Top ten markers associated with *Piscirickettsia salmonis* resistance defined as DD and BIN in coho salmon, using wssGBLUP method

**Ranking**	**Name**	**Chr***^**c**^*	**Pos (BP)***^**d**^*	**PEV***^**e**^*	**Genes***^**f**^*
**DD**					
**1**	**24987_127**	**11***^**a**^*	**30525921**	**5.302**	PIK3AP1, TIAL1, PCBD1
2	6135_83	3	37136738	3.225	NOXA1, UBAC1, KLHL20
3	7914_47	4	18281619	3.056	LRP5, NTRK3, KLHL25
4	25096_120	11	33047788	2.744	VA, INPP5A, CSAD
5	34697_43	15	32261231	2.081	CCDC153, KMT2A, LXN
6	52922_94	25	656773	1.802	NHLRC2, NRG3, LRRC4
7	41979_18	18	61818285	1.750	ROBO2, KCNJ1, LCE
8	22393_114	10	24996755	1.451	SLC34A2, SH3RF1, FYB
9	24553_70	11	19605173	1.294	VOS41, CHMP5, FAM49B
10	58185_41	29	22363292	1.211	TSC1, GFI1B, STOM
**BIN**					
**1**	**58185_41**	**29***^**a**^*	**22363292**	**4.270**	PHPT1, GSN, GS
**2**	**66451_65**	**31***^**a**^**^**b**^*	**12406**	**2.076**	PSMD14, CLDN10, CTSM
3	68326_79	31*^**b**^*	211676	2.073	ROBO1, PIK3CB, KCNJ1
4	45949_127	21	17289066	2.073	FLVCR1, VTA1, HIVEP2
5	36367_15	16	15005555	1.807	SEC24D, KACNIP4, MYOZ2
6	6135_83	3	37136738	1.291	NOXA1, UBAC1, KLHL20
7	37641_86	17	19982735	1.184	NR0B2, HIVEP3, EDN2
8	23665_61	10	55479897	1.170	HDAC5, CADM1, ICAM1
9	47149_112	22	17215562	1.133	PPARA, CDKN1B, KCNQ1
10	18750_95	8	26751149	1.094	GRID2, SMARCAD1, TSPAN3,

a Markers in common within the top ten along the four models.

b *Salmo salar* used as reference specie.

c Chromosome.

d Position in coho salmon reference genome.

e Percentage of Phenotypic variance.

f Summary of the genes located within 1-Mb window are in supporting information Table S4.

The availability of a high quality coho salmon reference genome (GenBank accession number MPVK00000000.1), made it possible to identify genes near this marker. Within ∼55 Kbp of this marker is the *phosphoinositide-3-kinase adaptor protein 1* (pik3ap1) gene; a gene related with innate host defense through B-cells development (Aiba *et al.* 2008; Herzog *et al.* 2009).

In the case of BIN, two molecular markers, explaining most of the genetic variance, were identified in all of the four models (Table 2). One of these markers is located on chromosome 29, while the second marker was identified only at a scaffold level (Scaffold04124) (Figure 3). For BIN, host immune response related genes were not found proximate to any of the suggestive molecular markers.

However, some genes within a 1-Mb window of these markers have been suggested to be involved with *P. salmonis* infection. The *14 KDa Phosphohistidine phosphatase-like* (PHPT1), *gelsolin-like* (GSN), and *glutamine synthase-like* (GS) genes are located on chromosome 29 and near associated markers. *Claudin-10* was found on scaffold04124. These genes have been previously identified as being up-regulated in *Salmo salar* individuals with low susceptibility to *P. salmonis (**Pulgar et al. 2015**)*. Moreover, the *retinoic acid receptor RXR-alpha-A-like* (RXRA) gene, located on chromosome 29, has previously been identified as a molecular biomarker for *P. salmonis* infections, and has been found to be down-regulated in macrophages during infection (Rise *et al.* 2004).

A full list of genes that are located within a 1-Mbp window proximate to the suggestive markers associated with *P. salmonis* resistance for Okis11, Okis29 and at a scaffold level, identified through wssGBLUP model, is shown in Table S4. In the case of the marker located on the scaffold, the surrounding sequence was blasted against the *Salmo salar* reference genome (NC_027300.1).

### Genetic parameters and predictions

Significant additive genetic variation was estimated for both DD and BIN when using all the data from challenged individuals from the 107 maternal, full-sib families (Table 1). Using the pedigree-based model without genomic data, estimates of the narrow sense heritability for DD and BIN were equal to 0.14 (± 0.034) and 0.27 (± 0.043), respectively.

**Figure 4:**
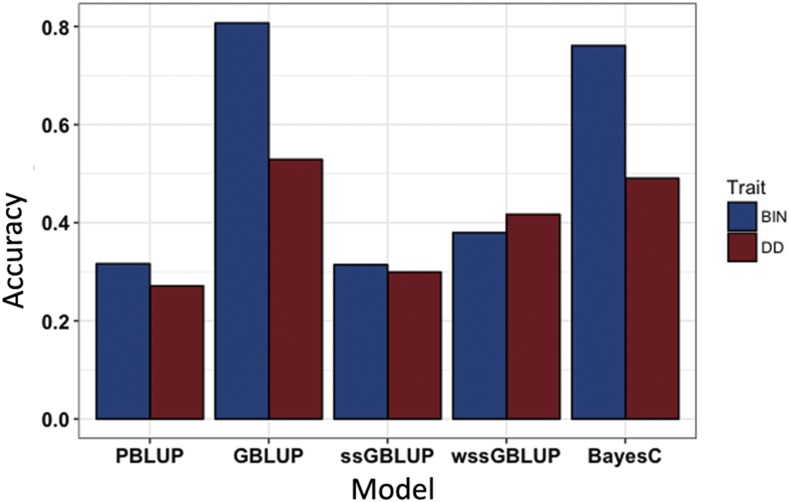
Comparison of predicted accuracies (R) for *Piscirickettsia salmonis* resistance in a coho salmon population comparing between PBLUP and models with genomic data for DD (red bars) and BIN (blue bars).

Based on a fivefold cross validation, the accuracy of the PBLUP model was slightly lower for DD (0.271) than for BIN (0.316) (Figure 4). When genomic data were included, accuracies for DD and BIN were higher than those achieved using only phenotypic data. However, there is considerable variation between models and trait definitions. The accuracies for the different models ranged from 0.299 (ssGBLUP) to 0.529 (GBLUP) for the DD trait, and from 0.314 (ssGBLUP) to 0.807 (GBLUP) for the BIN. For DD, all the models with genomic data outperformed the pedigree-based model. The relative increase in accuracy ranged from 10 (ssGBLUP) to 95% (GBLUP) (Figure 4). In the case of BIN, the relative increase in accuracy ranged from 20 (wssGBLUP) to 155% (GBLUP). However, one of the genomic models (ssGBLUP) had a similar accuracy to the PBLUP model, with a relative accuracy 1% lower than the pedigree-based model.

Interestingly, the accuracies obtained with GBLUP were higher than ssGBLUP and wssGBLUP for both traits. These accuracy values reached 0.529, 0.299 and 0.417 for DD and 0.807, 0.314 and 0.3797 for BIN. For both traits, the models with better performance were, GBLUP > Bayes C > wssGBLUP > ssGBLUP.

## Discussion

Significant genetic variation for *P. salmonis* resistance was detected in the present study. Moderate heritabilities were estimated using different trait definitions, either for DD or BIN. Estimated heritabilities were higher for resistance as a binary trait when compared to DD.

Previously, Yáñez *et al.* (2016a), estimated a heritability of 0.16 for resistance against *P. salmonis*, defined as day of death (from the same coho salmon population) through a bivariate linear model. Our study estimated a similar heritability value (0.14) in the same population. Differences in the estimations are likely due to the univariate model we used instead of a bivariate model. We also estimated heritability for *P. salmonis* resistance using a threshold model for the binary trait, which was higher (0.27) than the value reported for DD. These results are consistent with previous findings for resistance against *P. salmonis* in Atlantic salmon using pedigree information (Yáñez *et al.* 2013).

When resistance, defined as day of death, was analyzed using a linear model, heritability was estimated as 0.18 (0.03). When using a threshold model to analyze resistance as a binary trait, a heritability of 0.24 (0.04) was calculated (Yáñez *et al.* 2013, 2014b). The genetic variation and heritability values for *P. salmonis* resistance are in accord with different studies that have also found significant genetic resistance to other bacterial diseases in salmonid species (Gjøen *et al.* 1997; Ødegård *et al.* 2006; Vallejo *et al.* 2016).

Bacterial disease resistance has been suggested to be a polygenic trait in aquaculture species. For example, Palaiokostas *et al.* (2016) suggested that resistance against *Photobacterium damselae subsp*. has a polygenic architecture in Gilthead Sea Bream (*Sparus aurata*). Using a 50K SNP genotyping array, it was possible to elucidate a moderately polygenic architecture of *P. salmonis* resistance in Atlantic salmon (Correa *et al.* 2015b). In the current study, and using 9K SNPs, a similar genetic architecture for resistance against *P. salmonis* in coho salmon population was found. However, it is likely that the moderate number of individuals could limit the power to detect QTL of larger effect controlling *P. salmonis* resistance in coho salmon.

Among the top ten genetic markers for DD and BIN, one and two markers were identified among all four models, respectively. The availability of an annotated, coho salmon genome made it possible to identify the *phosphoinositide-3-kinase adaptor protein 1* (pik3ap1) (also known as the *adaptor protein B-cell PI3K adaptor* (BCAP)), a gene that is related with B-cell development (Herzog *et al.* 2009), proximate to the genetic marker found to be associated with resistance. The role of B-cells, through the humoral response, has been widely investigated and elucidated (reviewed in Janeway *et al.* 2001). B-cells may also have phagocytic activity in both rainbow trout (Li *et al.* 2006) and Atlantic salmon (Øverland *et al.* 2010). These cells are capable of ingesting large particles and bacteria; killing them through phagolysosome fusion (Li *et al.* 2006). Studies *in vitro*, showed that from the total phagocytic leukocytes isolated from Atlantic salmon head kidney, 37% were B-cells. Additionally, 77% were B-cells when leukocytes were isolated from peripheral blood (PB). The phagocytic ability of B-cells was three times higher than those observed in neutrophils in head kidney, while in PB no differences were observed (Øverland *et al.* 2010).

We hypothesize that B-cell development could help in the immune response against *P. salmonis* in coho salmon population through its phagocytic activity. Further studies are needed in order to have a better understanding of the role of these cells in the resistance against *P. salmonis*, and its ability to digest and kill bacteria. For resistance defined as the BIN trait, no genes related with immune response were identified near genetic markers associated with this trait. A possible explanation of this observation, is that the region near the genetic marker acts as a regulatory sequence. Also, it could be due to the relative small sample size for the GWAS, which ideally should be over 1000 animals (limiting the resolution of the GWAS).

The underlying genetic basis for this trait may have an important impact on the accuracy of genomic predictions. Thus, performance comparisons of different algorithms is required for either GS and GWAS when a complex trait is studied for the first time within a population (Vallejo *et al.* 2017b). The current study is the first to evaluate the genetic architecture of resistance against *Piscirickettsia salmonis* in coho salmon through different algorithms (to our knowledge). We used a genomic model that assumes that genetic variances are controlled by an infinite number of markers with minimum effects on the trait (*i.e.*, GBLUP). The GBLUP method calculates genomic relationship (G matrix) using all the genotyped markers for this reason. An extension of this method, which combines genomic (G) and pedigree-based (A) relationship information into the H relationship matrix (Aguilar *et al.* 2010; Legarra *et al.* 2014) was also evaluated. This method, called single-step GBLUP (ssGBLUP), mimics a Bayesian selection model in that it only fits SNPs that explain moderate to large genetic variance of the trait (Wang *et al.* 2012). Results of these different genomic-wide association analyses suggest that resistance against *Piscirickettsia* salmonis has a polygenic architecture, with no major QTL (*i.e.*, explaining >= 10% of the genetic variation) segregating in the current population (Figure 2 and Figure 3).

When resistance was defined as DD, the accuracy for the PBLUP model was slightly lower than resistance measured as BIN (0.271 and 0.316, respectively). These values are consistent with the results obtained for resistance against the bacterial disease pasteurellosis in *S. aurata* defined as day of death, authors reached an accuracy up to 0.30 through a pedigree-based model (Palaiokostas *et al.* 2016). However, both accuracy values are slightly lower when compared with values obtained for resistance against sea lice in Atlantic salmon. In this regard, Correa *et al.* (2017) reached an accuracy of 0.41 for resistance against *Caligus rogercresseyi*, while Tsai *et al.* (2016) reached a prediction accuracy ∼0.5 for resistance against *Lepeophtheirus salmonis* using PBLUP.

The results from our genomic predictions are in agreement with previous studies that showed higher estimated accuracies using GS than with PBLUP in the same half/full-sib family structure in salmon breeding programs (Nielsen *et al.* 2009; Lillehammer *et al.* 2013; Bangera *et al.* 2017).

The GS model that showed the best performance in terms of accuracy of predictions for DD was GBLUP. This was followed by Bayes C, wssGBLUP and ssGBLUP. The addition of genomic information allowed an improvement up to 95% in accuracy for this trait. The current study showed a relative accuracy prediction improvement of 54% when comparing PBLUP to wssGBLUP for this trait. This improvement is different to that obtained by Vallejo *et al.* (2016) for resistance against Bacterial Cold Water Disease (BCWD), defined as day of death, in rainbow trout (*Oncorhynchus mykiss*). The authors reported a reduction in the predictive ability (PA) of 20 and 26% using wssGBLUP, either with a chip array (40K) or through RAD sequencing (10K) respectively. The authors attributed this pronounced reduction to stochastic fluctuations due to a small training group of their study (n = 583). However, when the number of individuals was increased, this model outperformed the pedigree-based model, reaching a relative increase in accuracy up to 108%, for the same trait definition (Vallejo *et al.* 2017a).

In the case of BIN, the use of the ssGBLUP model, did not show an improvement in the accuracy prediction (with a reduction of 1% in comparison to the accuracy obtained though the pedigree-based model). However, there was an estimated relative increase in accuracy ranging from 20 to 155% when comparing PBLUP to the others genomic models. In this regard, Bayes C and GBLUP showed an increase in accuracy of 140% and 155%, respectively. These high values are similar to the improvements seen with resistance against BCWD defined as a binary trait, which reached a relative increase up to 97% (Vallejo *et al.* 2017a).

The relative improvements ranged in accuracy from -1 to 155% for BIN and from 10 to 95% for DD are greater to those obtained for other diseases resistance studies in Atlantic salmon; even with lower marker numbers. Using an identical random selection design as in the current study, sea lice resistance showed a relative improvement in accuracy of 22% relative to PBLUP when using 37K SNPs (Correa *et al.* 2017). For reliability, an improvement up to 52% with 220K SNPs was reached (Ødegård *et al.* 2014). Tsai *et al.* (2016) reported that when a non-full siblings design was used, an improvement in accuracy of 250% and 500% was reached in two different populations compared to PBLUP. However, when the methodology was changed to a random selection scheme this improvement only reached up to 27% using 33K SNPs.

In case of *Piscirickettsia salmonis* resistance, and using the same cross-validation scheme in our study, the relative reliability was increased by 25% and 30% for resistance, defined as day of death or as a binary trait, respectively with 50K SNPs (Bangera *et al.* 2017).

In the current study we evaluated a wide range of different models of GS for their potential implementation in aquaculture. The performance of each implemented model varied according to the underlying genetic architecture of the trait (Meuwissen *et al.* 2001; Daetwyler *et al.* 2010). Thus, it is valuable to perform these comparisons to identify the best performing method using real data. Similar accuracies among PBLUP and ssGLBUP models could be due to the predicted GEBV as both models are overrepresented by polygenic EBV (Bangera *et al.* 2017). GBLUP estimated genetic relationships using genotype and pedigree data rather than just average relationship as PBLUP (Habier *et al.* 2007). This allows a more accurate genetic relationship matrix and provides an increase in performance, as seen in the current data, due to the close family relationship. Moreover, GBLUP had significantly better performance when resistance was defined as a linear or binary trait compared to the other evaluated genomic models. We suggest that this could be an effect of only genotyping families from the opposite sides of the mortality distribution (*i.e.*, most resistant and most susceptible), and not all the challenged individuals. Bangera *et al.* (2017), reported similar accuracies among GBLUP and Bayesian methods between GBLUP and Bayes C using 10K SNPs in Atlantic salmon. Similar results that have been seen in dairy cattle for most traits (de Roos *et al.* 2009; Goddard 2009)

The greater improvement in accuracy for the binary trait BIN, compared with the linear trait DD, could be due a better fit of the threshold model for BIN than the fit of the linear model for DD. It could also be due to the higher estimated heritability.

We hypothesize that the large improvement values seen in the current study are likely due to an increased level of linkage disequilibrium (LD) found within this farmed coho salmon population. Additional studies are needed to elucidate the minimum number of markers necessary for GS.

Our results, using ddRAD sequencing, are in agreement with other genomic studies, which utilize GBS techniques with aquaculture species. Using RAD sequencing, some authors have previously performed genomic-wide association studies in rainbow trout looking for associations with disease resistance. From a total of 4K identified SNPs, 31 markers were significantly associated with either BCWD or Infectious Hematopoietic Necrosis Virus (IHNV) resistance as a binary trait (Campbell *et al.* 2014). These authors also showed the potential of these RAD markers to predict an animal’s phenotype. In the case of BCWD resistance, defined as a linear and binary trait, Palti *et al.* (2015b) identified suggestive and significant SNPs in two different families and candidate genes associated with this trait using ∼5K markers per family. Similar numbers of SNPs were used by Liu *et al.* (2015) to significantly associate 18 SNPs.

Genomic selection predictions are in accordance with studies aimed to evaluate GS using others GBS techniques, in both, relative increase in accuracy and number of discovered SNPs. Dou *et al.* (2016) predicted higher accuracies for shell height and shell width using GBLUP and Bayes methods in Yesso scallop (Patinopecten yessoensis) using 2K SNPs identified by 2b-RAD. Identical methodology allowed Palaiokostas *et al.* (2016) to reach a relative increase in accuracy up to 53% with 12K SNPs using Bayesian methods compared to PBLUP. A study in rainbow trout using RAD sequencing identified 10K SNPs. Even then, the accuracies were similar with GS models compared to PBLUP, and the authors predicted that increasing the number of individuals could lead to a relative increase in accuracy up to 69% (Vallejo *et al.* 2016).

The genotyping strategy was aimed at the i) evaluation of genomic selection methods; and ii) allowing the identification of molecular markers associated with the trait by means of GWAS. The aim was maximizing the phenotypic variance within the sample while keeping a balanced representation of fish per family. Thus, genotyping strategy was not totally random, but specifically focused on the most extreme families; 17 resistant and 16 susceptible families. We aimed at genotyping all the fish belonging to each selected family. Thus, each family was represented within the sample with an average of 23 (ranging from 11 to 43) fish/family.

The availability of dense SNP arrays for coho salmon, as it is already the case for Atlantic salmon (Houston *et al.* 2014; Yáñez *et al.* 2016b) and rainbow trout (Palti *et al.* 2015a), may increase the accuracy for predicting genomic breeding values and the power for the determination of the genetic factors involved in economically-important traits. It is also expected that in the near future, further functional studies for a better understanding of *P. salmonis* resistance and other complex traits in salmonids will be facilitated by the international initiative on the Functional Annotation of All Salmonid Genomes, FAASG (Macqueen *et al.* 2017).

We have evaluated different GS models, and demonstrated that the use of genomic prediction is a feasible strategy for the improvement of breeding value prediction. This information could be used for the implementation of genomic information in genetic programs for *Piscirickettsia salmonis* resistance in farmed coho salmon populations.

### Conclusions

Moderate significant genetic variation was estimated for resistance against *Piscirickettsia salmonis* in coho salmon, using either pedigree or genomic information. These results highlight the feasibility of including this trait into genetic improvement programs. Our study shows that genomic prediction methods, using ddRAD genotypes (including 9K SNPs), has a substantial advantage in terms of accuracy when compared to pedigree-based model for either DD or BIN. The improvement was up to 95 and 155% respectively in the current population. The association analyses were used to identify a gene related with B-cell development, which could also be involved in resistance against *P. salmonis*. To our knowledge, this is the first study aimed at dissecting the genetic architecture of *P. salmonis* resistance in a coho salmon population.

## Supplementary Material

Supplemental Material is available online at www.g3journal.org/lookup/suppl/doi:10.1534/g3.118.200053/-/DC1

## References

[bib1] Aguilar I. Misztal I. Johnson D. L. Legarra A. Tsuruta S., 2010 Hot topic: A unified approach to utilize phenotypic, full pedigree, and genomic information for genetic evaluation of Holstein final score. J. Dairy Sci. 93(2): 743–752. 10.3168/jds.2009-2730 20105546

[bib2] Aiba Y. Kameyama M. Yamazaki T. Tedder T. F. Kurosaki T., 2008 Regulation of B-cell development by BCAP and CD19 through their binding to phosphoinositide 3-kinase. Blood 111(3): 1497–1503. 10.1182/blood-2007-08-109769 18025150

[bib3] Baird N. A. Etter P. D. Atwood T. S. Currey M. C. Shiver A. L., 2008 Rapid SNP discovery and genetic mapping using sequenced RAD markers. PLoS One 3(10): e3376 10.1371/journal.pone.0003376 18852878 PMC2557064

[bib4] Bangera R. Correa K. Lhorente J. P. Figueroa R. Yáñez J. M., 2017 Genomic predictions can accelerate selection for resistance against Piscirickettsia salmonis in Atlantic salmon (Salmo salar). BMC Genomics 18(1): 121 10.1186/s12864-017-3487-y 28143402 PMC5282740

[bib5] Brieuc M. S. O. Waters C. D. Seeb J. E., and K. A. Naish, 2014 A dense linkage map for Chinook salmon (*Oncorhynchus tshawytscha*) reveals variable chromosomal divergence after an ancestral whole genome duplication event. G3 Genes Genomes Genet. 4: 447–460. https://doi.org/10.1534/g3.113.009316 10.1534/g3.113.009316 PMC3962484 24381192

[bib6] Campbell N. R. LaPatra S. E. Overturf K. Towner R. Narum S. R., 2014 Association mapping of disease resistance traits in rainbow trout using restriction site associated DNA sequencing. G3 Genes Genomes Genet. 4: 2473–81. https://doi.org/10.1534/g3.114.014621 10.1534/g3.114.014621 PMC4267942 25354781

[bib7] Catchen J. M. Amores A. Hohenlohe P. Cresko W. Postlethwait J. H., 2011 *Stacks*: building and genotyping loci *de novo* from short-read sequences. G3 Genes Genomes Genet. 1: 171–182. 10.1534/g3.111.000240 PMC3276136 22384329

[bib8] Catchen J. Hohenlohe P. A. Bassham S. Amores A. Cresko W. A., 2013 Stacks: An analysis tool set for population genomics. Mol. Ecol. 22(11): 3124–3140. 10.1111/mec.12354 23701397 PMC3936987

[bib9] Correa K. Bangera R. Figueroa R. Lhorente J. P. Yáñez J. M., 2017 The use of genomic information increases the accuracy of breeding value predictions for sea louse (Caligus rogercresseyi) resistance in Atlantic salmon (Salmo salar). Genet. Sel. Evol. 49(1): 15 10.1186/s12711-017-0291-8 28143593 PMC5282780

[bib10] Correa K. Filp M. Cisterna D. Cabrejos M. E. Gallardo-Escárate C., 2015 a Effect of triploidy in the expression of immune-related genes in coho salmon Oncorhynchus kisutch (Walbaum) infected with Piscirickettsia salmonis. Aquacult. Res. 46: 59–63. 10.1111/are.12584

[bib11] Correa K. Lhorente J. Lopez M. Bassini L. Naswa S., 2015 b Genome-wide association analysis reveals loci associated with resistance against Piscirickettsia salmonis in two Atlantic salmon (Salmo salar L.) chromosomes. BMC Genomics 16(1): 854 10.1186/s12864-015-2038-7 26499328 PMC4619534

[bib12] Cvitanich J. Garate O. Smith C. E., 1991 The isolation of a rickettsia‐like organism causing disease and mortality in Chilean salmonids and its confirmation by Koch’s postulate. J. Fish Dis. 14(2): 121–145. 10.1111/j.1365-2761.1991.tb00584.x

[bib13] Daetwyler H. D. Pong-Wong R. Villanueva B. Woolliams J. A., 2010 The impact of genetic architecture on genome-wide evaluation methods. Genetics 185(3): 1021–1031. 10.1534/genetics.110.116855 20407128 PMC2907189

[bib14] Davey J. W. Cezard T. Fuentes-Utrilla P. Eland C. Gharbi K., 2013 Special features of RAD Sequencing data: Implications for genotyping. Mol. Ecol. 22(11): 3151–3164. 10.1111/mec.12084 23110438 PMC3712469

[bib15] Dou J. Li X. Fu Q. Jiao W. Li Y., 2016 Evaluation of the 2b-RAD method for genomic selection in scallop breeding. Sci. Rep. 6(1): 19244 10.1038/srep19244 26754638 PMC4709697

[bib16] Dufflocq P. Lhorente J. P. Bangera R. Neira R. Newman S., 2016 Correlated response of flesh color to selection for harvest weight in coho salmon (Oncorhynchus kisutch). Aquaculture 472: 6–11.

[bib17] Elshire R. J. Glaubitz J. C. Sun Q. Poland J. A. Kawamoto K., 2011 A robust, simple genotyping-by-sequencing (GBS) approach for high diversity species. PLoS One 6(5): e19379 10.1371/journal.pone.0019379 21573248 PMC3087801

[bib18] Falconer D.S. T.F.C. Mackay, 1996 Introduction to Quantitative Genetics. fourth ed. Longman Group Limited, Harlow, Essex, U.K.

[bib19] FAO, 2016 Fisheries and Aquaculture Department [Online]. (Rome. Updated 31 January 2016. http://www.fao.org/fishery/statistics/global-aquaculture-production/query/en

[bib20] Fernando R.L. Garrick D., 2013 Bayesian Methods Applied to GWAS. In: Gondro C., van der Werf J., Hayes B. (eds) Genome-Wide Association Studies and Genomic Prediction. Methods in Molecular Biology (Methods and Protocols), vol 1019 Humana Press, Totowa, NJ. 10.1007/978-1-62703-447-0_10 23756894

[bib21] Fernando R. L. Habier D. Stricker C. Dekkers J. C. M. Totir L. R., 2007 Genomic selection. Acta Agric. Scand. Sect. Anim. Sci. 57: 192–195. 10.1080/09064700801959395

[bib22] Gjøen H. M. Refstie T. Ulla O. Gjerde B., 1997 Genetic correlations between survival of Atlantic salmon in challenge and field tests. Aquaculture 158(3-4): 277–288. 10.1016/S0044-8486(97)00203-2

[bib23] Goddard M., 2009 Genomic selection: Prediction of accuracy and maximisation of long term response. Genetica 136(2): 245–257. 10.1007/s10709-008-9308-0 18704696

[bib24] Gonen S. Lowe N. R. Cezard T. Gharbi K. Bishop S. C., 2014 Linkage maps of the Atlantic salmon (Salmo salar) genome derived from RAD sequencing. BMC Genomics 15(1): 166 10.1186/1471-2164-15-166 24571138 PMC4028894

[bib25] Habier D. Fernando R. L. Dekkers J. C. M., 2007 The impact of genetic relationship information on genome-assisted breeding values. Genetics 177: 2389–2397. 10.1534/genetics.107.081190 18073436 PMC2219482

[bib26] Habier D. Fernando R. L. Kizilkaya K. Garrick D. J. Meuwissen T., 2011 Extension of the bayesian alphabet for genomic selection. BMC Bioinformatics 12(1): 186 10.1186/1471-2105-12-186 21605355 PMC3144464

[bib27] Hayes B. Goddard M., 2010 Genome-wide association and genomic selection in animal breeding. Genome 53(11): 876–883. 10.1139/G10-076 21076503

[bib28] Herzog S. Reth M. Jumaa H., 2009 Regulation of B-cell proliferation and differentiation by pre-B-cell receptor signalling. Nat. Rev. Immunol. 9(3): 195–205. 10.1038/nri2491 19240758

[bib29] Houston R. D. Davey J. W. Bishop S. C. Lowe N. R. Mota-Velasco J. C., 2012 Characterisation of QTL-linked and genome-wide restriction site-associated DNA (RAD) markers in farmed Atlantic salmon. BMC Genomics 13(1): 244 10.1186/1471-2164-13-244 22702806 PMC3520118

[bib30] Houston R. D. Taggart J. B. Cézard T. Bekaert M. Lowe N. R., 2014 Development and validation of a high density SNP genotyping array for Atlantic salmon (Salmo salar). BMC Genomics 15(1): 90 10.1186/1471-2164-15-90 24524230 PMC3923896

[bib31] Janeway C. Travers P. Walport M. Shlomchik M., 2001 B-cell activation by armed helper T cells., pp. 343–360 in *Immunobiology 5*, *The Immune System in Health and Disease*, Ed. 5 Churchill Livingstone, New York.

[bib32] Legarra A. Christensen O. F. Aguilar I. Misztal I., 2014 Single Step, a general approach for genomic selection. Livest. Sci. 166: 54–65. 10.1016/j.livsci.2014.04.029

[bib33] Legarra A. Robert-Granié C. Manfredi E. Elsen J.-M., 2008 Performance of genomic selection in mice. Genetics 180: 611–618. https://doi:10.1534/genetics.108.088575 18757934 10.1534/genetics.108.088575 PMC2535710

[bib34] Li J. Barreda D. R. Zhang Y.-A. Boshra H. Gelman A. E., 2006 B lymphocytes from early vertebrates have potent phagocytic and microbicidal abilities. Nat. Immunol. 7(10): 1116–1124. 10.1038/ni1389 16980980

[bib35] Li H. Durbin R., 2009 Fast and accurate short read alignment with Burrows-Wheeler transform. Bioinformatics 25(14): 1754–1760. 10.1093/bioinformatics/btp324 19451168 PMC2705234

[bib36] Lillehammer M. Meuwissen T. H. E. Sonesson A. K., 2013 A low-marker density implementation of genomic selection in aquaculture using within-family genomic breeding values. Genet. Sel. Evol. 45(1): 39 10.1186/1297-9686-45-39 24127852 PMC3854107

[bib37] Liu S. Vallejo R. L. Palti Y. Gao G. Marancik D. P., 2015 Identification of single nucleotide polymorphism markers associated with bacterial cold water disease resistance and spleen size in rainbow trout. Front. Genet. 6: 1–10. 10.3389/fgene.2015.00298 26442114 PMC4585308

[bib38] Lynch M. Walsh B., 1998 Genetics and analysis of quantiative traits, Sinauer Associates, Sunderland.

[bib39] Macqueen D. J. Primmer C. R. Houston R. D. Nowak B. F. Bernatchez L., 2017 Functional Annotation of All Salmonid Genomes (FAASG): an international initiative supporting future salmonid research, conservation and aquaculture. BMC Genomics 18: 484 10.1186/s12864-017-3862-8 28655320 PMC5488370

[bib40] Meuwissen T. H. Hayes B. J. Goddard M. E., 2001 Prediction of total genetic value using genome-wide dense marker maps. Genetics 157: 1819–1829. 11290733 10.1093/genetics/157.4.1819 PMC1461589

[bib41] Misztal, I., S. Tsuruta, D. Lourenco, Y. Masuda, I. Aguilar *et al.,* 2016 Manual for BLUPF90 Family of Programs. University of Georgia, Athens, GA.

[bib42] Moen T. Torgersen J. Santi N. Davidson W. S. Baranski M., 2015 Epithelial cadherin determines resistance to infectious pancreatic necrosis virus in Atlantic salmon. Genetics 200: 1313–1326. https://doi: 10.1534/genetics.115.175406 26041276 10.1534/genetics.115.175406 PMC4574245

[bib43] Nielsen H. M. Sonesson A. K. Yazdi H. Meuwissen T. H. E., 2009 Comparison of accuracy of genome-wide and BLUP breeding value estimates in sib based aquaculture breeding schemes. Aquaculture 289(3-4): 259–264. 10.1016/j.aquaculture.2009.01.027

[bib44] Ødegård J. Baranski M. Gjerde B. Gjedrem T., 2011 Methodology for genetic evaluation of disease resistance in aquaculture species: Challenges and future prospects. Aquacult. Res. 42: 103–114. 10.1111/j.1365-2109.2010.02669.x

[bib45] Ødegård J. Moen T. Santi N. Korsvoll S. A. Kjøglum S., 2014 Genomic prediction in an admixed population of Atlantic salmon (*Salmo salar*). Front. Genet. 5: 1–8. https://doi: 10.3389/fgene.2014.00402 25484890 10.3389/fgene.2014.00402 PMC4240172

[bib46] Ødegård J. Olesen I. Gjerde B. Klemetsdal G., 2006 Evaluation of statistical models for genetic analysis of challenge test data on furunculosis resistance in Atlantic salmon (Salmo salar): Prediction of field survival. Aquaculture 259(1-4): 116–123. 10.1016/j.aquaculture.2006.05.034

[bib47] Øverland H. S. Pettersen E. F. Rønneseth A. Wergeland H. I., 2010 Phagocytosis by B-cells and neutrophils in Atlantic salmon (Salmo salar L.) and Atlantic cod (Gadus morhua L.). Fish Shellfish Immunol. 28(1): 193–204. 10.1016/j.fsi.2009.10.021 19874896

[bib48] Palaiokostas C. Ferarreso S. Franch R. Houston R. D. Bargelloni L., 2016 Genomic prediction of resistance to pasteurellosis in gilthead sea bream (*Sparus aurata*) using 2b-RAD sequencing. G3 Genes Genomes Genet. Available at: https://doi:10.1534/g3.116.035220 10.1534/g3.116.035220 PMC5100868 27652890

[bib49] Palti Y. Gao G. Liu S. Kent M. P. Lien S., 2015 a The development and characterization of a 57K single nucleotide polymorphism array for rainbow trout. Mol. Ecol. Resour. 15(3): 662–672. 10.1111/1755-0998.12337 25294387

[bib50] Palti Y. Vallejo R. L. Gao G. Liu S. Hernandez A. G., 2015 b Detection and validation of QTL affecting bacterial cold water disease resistance in rainbow trout using restriction-site associated dna sequencing. PLoS One 10(9): e0138435 10.1371/journal.pone.0138435 26376182 PMC4574402

[bib51] Peterson B. K. Weber J. N. Kay E. H. Fisher H. S. Hoekstra H. E., 2012 Double digest RADseq: An inexpensive method for *de novo* SNP discovery and genotyping in model and non-model species. PLoS One 7: e37135 https://doi:10.1371/journal.pone.0037135 22675423 10.1371/journal.pone.0037135 PMC3365034

[bib52] Pulgar R. Hödar C. Travisany D. Zuñiga A. Domínguez C., 2015 Transcriptional response of Atlantic salmon families to Piscirickettsia salmonis infection highlights the relevance of the iron-deprivation defence system. BMC Genomics 16(1): 495 10.1186/s12864-015-1716-9 26141111 PMC4490697

[bib53] Rise M. L. Jones S. R. M. Brown G. D. von Schalburg K. R. Davidson W. S., 2004 Microarray analyses identify molecular biomarkers of Atlantic salmon macrophage and hematopoietic kidney response to Piscirickettsia salmonis infection. Physiol. Genomics 20(1): 21–35. 10.1152/physiolgenomics.00036.2004 15454580

[bib54] Robledo D. Palaiokostas C. Bargelloni L. Martínez P. Houston R., 2017 Applications of genotyping by sequencing in aquaculture breeding and genetics. Rev. Aquac. 1–13. 10.1111/raq.12193 PMC6128402 30220910

[bib55] de Roos A. P. W. Hayes B. J. Goddard M. E., 2009 Reliability of genomic predictions across multiple populations. Genetics 183(4): 1545–1553. 10.1534/genetics.109.104935 19822733 PMC2787438

[bib56] Rozas M. Enríquez R., 2014 Piscirickettsiosis and Piscirickettsia salmonis in fish: a review. J. Fish Dis. 37(3): 163–188. 10.1111/jfd.12211 24279295

[bib57] Sernapesca, 2016 Informe Sanitario de Salmonicultura en Centros Marinos 2016. ([Online]. Valparaiso, Chile. Updated 14 August 2017. http://www.sernapesca.cl/index.php?option=com_remository&Itemid=246&func=fileinfo&id=27560).

[bib58] Stear M. J. Bishop S. C. Mallard B. A. Raadsma H., 2001 The sustainability, feasibility and desirability of breeding livestock for disease resistance. Res. Vet. Sci. 71(1): 1–7. 10.1053/rvsc.2001.0496 11666141

[bib59] Tsai H.-Y. Hamilton A. Tinch A. E. Guy D. R. Bron J. E., 2016 Genomic prediction of host resistance to sea lice in farmed Atlantic salmon populations. Genet. Sel. Evol. 48(1): 47 10.1186/s12711-016-0226-9 27357694 PMC4926294

[bib60] Vallejo R. L. Leeds T. D. Fragomeni B. O. Gao G. Hernandez A. G., 2016 Evaluation of genome-enabled selection for bacterial cold water disease resistance using progeny performance data in rainbow trout: insights on genotyping methods and genomic prediction models. Front. Genet. 7: 1–13. 10.3389/fgene.2016.00096 27303436 PMC4883007

[bib61] Vallejo R. L. Leeds T. D. Gao G. Parsons J. E. Martin K. E., 2017 a Genomic selection models double the accuracy of predicted breeding values for bacterial cold water disease resistance compared to a traditional pedigree-based model in rainbow trout aquaculture. Genet. Sel. Evol. 49(1): 17 10.1186/s12711-017-0293-6 28148220 PMC5289005

[bib62] Vallejo R. L. Liu S. Gao G. Fragomeni B. O. Hernandez A. G., 2017 b Similar genetic architecture with shared and unique quantitative trait loci for bacterial cold water disease resistance in two rainbow trout breeding populations. Front. Genet. 8: 156 10.3389/fgene.2017.00156 29109734 PMC5660510

[bib63] VanRaden P. M., 2008 Efficient methods to compute genomic predictions. J. Dairy Sci. 91(11): 4414–4423. 10.3168/jds.2007-0980 18946147

[bib64] Wang H. Misztal I. Aguilar I. Legarra A. Fernando R. L., 2014 Genome-wide association mapping including phenotypes from relatives without genotypes in a single-step (ssGWAS) for 6-week body weight in broiler chickens. Front. Genet. 5: 1–10. 10.3389/fgene.2014.00134 24904635 PMC4033036

[bib65] Wang H. Misztal I. Aguilar I. Legarra A. Muir W. M., 2012 Genome-wide association mapping including phenotypes from relatives without genotypes. Genet. Res. 94(02): 73–83. 10.1017/S0016672312000274 22624567

[bib66] Yáñez J. M. Bangera R. Lhorente J. P. Barria A. Oyarzun M., 2016 a Negative genetic correlation between resistance against Piscirickettsia salmonis and harvest weight in coho salmon (Oncorhynchus kisutch). Aquaculture 459: 8–13. 10.1016/j.aquaculture.2016.03.020

[bib67] Yáñez J. M. Naswa S. Lopez M. E. Bassini L. Correa K., 2016 b Genomewide single nucleotide polymorphism discovery in Atlantic salmon (Salmo salar): validation in wild and farmed American and European populations. Mol. Ecol. Resour. 16(4): 1002–1011. 10.1111/1755-0998.12503 26849107

[bib68] Yáñez J. M. Bangera R. Lhorente J. P. Oyarzún M. Neira R., 2013 Quantitative genetic variation of resistance against Piscirickettsia salmonis in Atlantic salmon (Salmo salar). Aquaculture 414–415: 155–159. 10.1016/j.aquaculture.2013.08.009

[bib69] Yáñez J. M. Bassini L. N. Filp M. Lhorente J. P. Ponzoni R. W., 2014 a Inbreeding and effective population size in a coho salmon (Oncorhynchus kisutch) breeding nucleus in Chile. Aquaculture 420–421: S15–S19. 10.1016/j.aquaculture.2013.05.028

[bib70] Yáñez J. M. Houston R. D. Newman S., 2014 b Genetics and genomics of disease resistance in salmonid species. Front. Genet. 5: 1–13. https://doi:10.3389/fgene.2014.00415 25505486 10.3389/fgene.2014.00415 PMC4245001

[bib71] Yáñez J. M. Lhorente J. P. Bassini L. N. Oyarzún M. Neira R., 2014 c Genetic co-variation between resistance against both Caligus rogercresseyi and Piscirickettsia salmonis, and body weight in Atlantic salmon (Salmo salar). Aquaculture 433: 295–298. 10.1016/j.aquaculture.2014.06.026

[bib73] Yoshida G.M. Bangera R. Carvalheiro R. Correa K. Figueroa R. Lhorente J. P. Yáñez J. M., 2017 Genomic prediction accuracy for resistance against *Piscirickettsia salmonis* in farmed rainbow trout. G3 Genes Genomes Genet. 8:719–726. https://doi.org/10.1534/g3.117.300499 10.1534/g3.117.300499 PMC5919750 29255117

