# Supplementary material for: Genomic Predictions and Genome-Wide Association Study of Resistance Against Piscirickettsia salmonis in Coho Salmon (Oncorhynchus kisutch) Using ddRAD Sequencing
Source: G3 (Bethesda). 2018 Feb 22;8(4):1183–94. doi: 10.1534/g3.118.200053 (PMC5873909; doi:10.1534/g3.118.200053)
Supplement: Supplementary file 3 [file 1183TableS4.docx]

**Table S4.** Full list of genes located within top ten 1-Mb windows associated with P. salmonis resistance in coho salmon, for DD and BIN.

| CHR_WIN | Genes^1,2^ |
| --- | --- |
| Resistance as Day of Death (DD) | |
| 24987_127 | *SULT3A1, BAG3, CACUL1, CSGALNACT2, EIF3A, EMX2, MCMBP, ABHD12, MYPN, TIAL1, PDLIM1,INPP5F,PIK3AP1, PCBP3, FAM204A,FAM45A,RET, PCBD1,,RAB11FIP2, SEC23IP,SAP, SFXN4, SORBS1,SGPL1, PRDX3, TM9SF3 unc LOC109899060, unc LOC109899061, unc LOC109899063, VWC2L* |
| 6135_83 | *DFNB31, NOXA1, CAMSAP1, UBAC1, KCNT1, KLHL20, COL27A1, FABG, LRRC8A, FAM129B, NPDC1, FUT7, ABCA2, DPH7, PAXX, IPO11, unc LOC109872505, unc LOC109883354* |
| 7914_47 | *NTRK3, KLHL25, SV2B, AKAP13, LRP5, unc LOC109888557, unc LOC109889240* |
| 25096_120 | *NKX6-2, INPP5D, CSAD, TDCB, PLAC9, TMEM254, ADGRA3, GABRP, CFAP46, STK32C, PWWP2B, unc LOC109899071, unc LOC109898833, unc LOC109899629, unc LOC109899622, VA* |
| 34697_43 | *CCDC153, CBL, MCAM, RNF26, C1QTNF5, KMT2A, UBE4A, PDZK1, ABCG4, PBGD, ITPKC, ARCN1, LXN, SNRPA, ARHGEF12* |
| 52922_94 | *NHLRC2, NRG3, KCNC1, PPFIA3, LRRC4, USP31, ABLIM1, unc LOC109870082* |
| 41979_18 | *ROBO2, KCNJ1, LCE* |
| 22393_114 | *SLC34A2, PROCPAS6, PRDM5, CPEB2, TRIM16, FBXL5, CBR4, DAB1, FYB, PLPP3, USP24, BLOC1S2, BSND1, DHCR24TMEM27, PSMB5, CDH24, SH3RF1, NDNF, CqQTNF7 PRKAA2, unc LOC109897645, unc LOC109898001* |
| 24553_70 | *VPS41, TTN, NKTR, SOX17, LYPLA1, MRPL15, RGS20, LPIN2, MYOM2, EMILIN2, TRIM16, CHMP5, EEF1A1, FAM49B, ANKH, MYC, YAE1D1, POU6F2, METTL4, MYL9, FASTKD3, SMCHD1, unc LOC10989948, unc LOC109899467* |
| 58185_41 | *ENTPD2, DAF36, GTF3C5, TSC1, GTF3C5, GRIN1, GFI1B, FABP1, SMYD1, SPR, VT, LZTS1, FASTKD5, YTHDC2, MCC, H2, MYO3B, ARRDC1, MAMDC4, MALRD1, PHPT1, GTF3C4, BARX1, STOM, GSN, CEL, unc LOC109874322, unc LOC109873509* |
| Resistance as a Binary trait (BIN) | |
| 58185_41 | *PHPT1, MAMDC4,ARRDC1, BARX1, MEOX1,BAL,H2, DAF36, MCC, ENTPD2,STOM,GSN, GTF3C4,GTF3C5, IGLURS, NMDA 1,GS, TSC1, MALRD1, MYO3B, RXRA, unc LOC109873509, unc LOC109873509, unc LOC109873523, unc LOC109874318, unc LOC109874322* |
| 66451_65^3^ | *OXGR1, PSMD14, ADCY10, ATP5B, B3GALT1, BSEP, CASQ2, CTSM, CLDN10,CLDN10A, COL28A1, TIMMDC1, CSRNP3,DPP4,FRMPD4, GEMIN8, GCG2, GCA,HS6ST3, KLHL41,MOSPD2,MSL3,ABCC4, GPM6B,PRPS2,KCNH7,RAB9A,RDH7, SCN2A,SLC4A10, TBR1, unc LOC106575284, unc LOC106575285, unc LOC106575292, unc LOC106575299, unc LOC106575332, unc LOC106575333, unc LOC106575343, XIRP2, DZIP1* |
| 68326_79^3^ | *ROBO1, ROBO2, PIK3CB, FOXL2, KCNJ1, unc LOC106613195* |
| 45949_127 | *FLVCR1, VASH2, GALNT14, EHD3, STUM, GJE1, VTA1, NMBR, ADGRG6, EDN1, HIVEP2, AIG1, ITPKB, ATF3, TATDN1, BATF, COQ8A, unc LOC109866141, unc LOC109866401, unc LOC109866631, unc LOC109866509, unc LOC10986614, unc LOC109866275, unc LOC109866401* |
| 36367_15 | *SEC24D, KCNIP4, USP53, MYOZ2, SYNPO2, ADGRA3, GBA3, unc LOC109906516* |
| 6135_83 | *DFNB31, NOXA1, CAMSAP1, UBAC1, KCNT1, KLHL20, COL27A1, FABG, LRRC8A, FAM129B, NPDC1, FUT7, ABCA2, DPH7, PAXX, IPO11, unc LOC109872505, unc LOC109883354* |
| 37641_86 | *NTLA, NR0B2, NUDC, KDF1, COL22A1, HIVEP3, EDN2, PHACTR4, OPRD1, COL9A1, FAM135B, unc LOC109908692, unc LOC109908508, unc LOC109907293* |
| 23665_61 | *EP300, CBX7, DESI1, SLIT2, LSM12, HDAC5, CYB561, TANC2, RAVER1, CDC37, CADM1, ICAM1, HMCN1, S1PR2, DNMT1, EIF3G, P2RY11, PPAN, ANGPTL2, MFAP3, TRIP10, ACO2, TOB2, TEF, RANGAP1, CHADL, RBX1, ST13, ZC3H7B* |
| 47149_112 | *PPARA, YARS2, CDKN1B, KCNQ1, MRPS35, MANSC4, ZSCAN2, TEAD4, DENND2A, PPM1H, FBLN1, MON2, SLC6A13, AKR1D1, WNT7B* |
| 18750_95 | *GRID2, ATOH1, SMARCAD1, MAPK4, TSPAN3, GKAP1, ARRDC3, GPR98, TRIM36, CCSER1, FEM1C, TMED7, CDO1, AP3S1* |

Unc. – Uncharacterized

^1^Suggestive genes located 1-Mb window.

*^2^SCoho salmon* used as reference species.

^3^Salmo salar used as reference genome
